# Sequential detection of influenza epidemics by the Kolmogorov-Smirnov test

**DOI:** 10.1186/1472-6947-12-112

**Published:** 2012-10-03

**Authors:** Pau Closas, Ermengol Coma, Leonardo Méndez

**Affiliations:** 1 Centre Tecnològic de Telecomunicacions de Catalunya (CTTC), Av. Carl Friedrich Gauss 7, 08860 Castelldefels, Barcelona, Spain; 2 Institut Català de la Salut (ICS), Sistema d’Informació dels Serveis d’Atenció Primària (SISAP), Gran Via de les Corts Catalanes, 587-589, 08007 Barcelona, Spain

**Keywords:** Influenza, Sequential methods, Statistical test, Detection theory

## Abstract

**Background:**

Influenza is a well known and common human respiratory infection, causing significant morbidity and mortality every year. Despite Influenza variability, fast and reliable outbreak detection is required for health resource planning. Clinical health records, as published by the Diagnosticat database in Catalonia, host useful data for probabilistic detection of influenza outbreaks.

**Methods:**

This paper proposes a statistical method to detect influenza epidemic activity. Non-epidemic incidence rates are modeled against the exponential distribution, and the maximum likelihood estimate for the decaying factor *λ *is calculated. The sequential detection algorithm updates the parameter as new data becomes available. Binary epidemic detection of weekly incidence rates is assessed by Kolmogorov-Smirnov test on the absolute difference between the empirical and the cumulative density function of the estimated exponential distribution with significance level 0 ≤* α *≤ 1.

**Results:**

The main advantage with respect to other approaches is the adoption of a statistically meaningful test, which provides an indicator of epidemic activity with an associated probability. The detection algorithm was initiated with parameter *λ*_0 _= 3.8617 estimated from the training sequence (corresponding to non-epidemic incidence rates of the 2008-2009 influenza season) and sequentially updated. Kolmogorov-Smirnov test detected the following weeks as epidemic for each influenza season: 50−10 (2008-2009 season), 38−50 (2009-2010 season), weeks 50−9 (2010-2011 season) and weeks 3 to 12 for the current 2011-2012 season.

**Conclusions:**

Real medical data was used to assess the validity of the approach, as well as to construct a realistic statistical model of weekly influenza incidence rates in non-epidemic periods. For the tested data, the results confirmed the ability of the algorithm to detect the start and the end of epidemic periods. In general, the proposed test could be applied to other data sets to quickly detect influenza outbreaks. The sequential structure of the test makes it suitable for implementation in many platforms at a low computational cost without requiring to store large data sets.

## Background

Influenza is a well known and common human respiratory infection. It is responsible of significant morbidity and mortality every year. The World Health Organization (WHO) estimates that annual epidemics result in about 3 to 5 million cases of severe illness and about 250,000 to 500,000 casualties worldwide [1]. Influenza has a seasonal pattern and in the Northern hemisphere epidemics occurs between November and March.

Sentinel networks covering less than 2% of the population have been the traditional surveillance system. More recently, electronic health records are widely implemented in some regions making available a significant amount of health related data. In Catalonia, primary care doctors have been routinely registering their activity in eCAP (an electronic health recording system) since 2006. This accounts for over 3,500 physicians collecting data of nearly 6 million people (80% of the population) [2,3]. The data produced is a key source of information that could also be used for surveillance of certain diseases. In 2011 SISAP (the catalan acronym for Information Systems for Primary Care Services) developed a process of extracting health conditions data from eCAP in order to provide information about some diseases. Nowadays, SISAP publishes weekly information about all cases of those infectious diseases, available on Diagnosticat ( http://4.sisap.cat/diagnosticat).

Among the large variety of tracked diseases, we focus on influenza. Influenza data on Diagnosticat has shown its validity as compared to the sentinel network data. Its main advantage is that its data is available faster [3], and is thus theoretically better suited for timely activation of emergency responses. More precisely, we are interested in the outbreak detection of such disease by means of a statistical surveillance system [4]. The objective is to provide an indicator as soon as data gives enough evidence to assess the event. Moreover, we seek to apply the developed algorithms to the Diagnosticat’s database. Other influenza surveillance systems reported in the literature are briefly commented in the following. In [5], the authors presented an online tool to analyze epidemiological data. They used non-epidemic training data to fit a time-series model for the periodic baseline level, thus following a parametric approach for their purposes. [6] proposed a method to detect deviations from the baseline that was based on fitting historical data to a time-series regression model. Time-series analysis were also used in the same context in [7]. A weighted moving average algorithm was considered in [8] to monitor the time-series. In contrast, in the present work we use non-epidemic data to infer the parameters of the probability density function of non-epidemic periods. Therefore, we make no assumptions on the time evolution of the disease and instead we resort to a probabilistic characterization. The work in [9] suggests that non-parametric methods are best suited to influenza modeling, due to its large variability between years. The authors of [9] focused on other features of the influenza cycle rather than disease outbreak, such as the peak after which the influenza incidence starts to decline, and applied the method to a case study in Sweden. More involved methods, combining both approaches, are possible. In [10] a switching Markov model was used with an autoregressive process to model epidemic data and a white Gaussian process for non-epidemic modeling. This method was implemented in a web-based application developed by the same authors and presented in [11]. Other references point to imaginative, yet reliable methods to detect possible epidemic outbreaks. For instance, in [12], Google search queries were used to track influenza-like illness in a population. In a similar fashion, Twitter was used in [13].

This paper presents a statistical surveillance system which provides an automated detection of influenza epidemics. Health resource planning is an application which could benefit from this tool. The proposed method is able to operate on-line^a^ and it is based on the statistical characterization of non-epidemic influenza incidence rates. A major advantage of this approach is its statistical meaningfulness, and thus detection is not only a binary result but the confidence in its outcome can be assessed in terms of probabilities resorting to hypothesis testing theory. Non-epidemic data is modeled with an exponential distribution, in the vein of [14]. The unique parameter of the distribution is the decaying factor, that is initially estimated by a training data set and sequentially updated as new observations are recorded. Such statistical characterization is used to design a detector based on the one-sample Kolmogorov-Smirnov test. The method was applied to data in the Diagnosticat database to successfully detect influenza activity.

The remainder of the paper is as follows. Diagnosticat is introduced in the following section Then we provide insights on the statistical distribution of influenza incidence rates, as well as how the relevant parameters can be estimated from the observations. The general statistical detector is proposed, and the results for the catalan case study are presented.

## Methods

### Diagnosticat: an open epidemiological database

Diagnosticat is an open-access database which contains reports of many diseases occurring in Catalonia, such as influenza, papilloma or chickenpox. The information available in the Diagnosticat database includes all clinical influenza diagnoses codes (ICD-10 code) and is obtained weekly from eCAP through an automated process. The website is timely updated a few minutes after every finished epidemiological week (EW). After the extraction, a computer algorithm automatically creates the different tables with the information that is used in the website. No identifiable or personal information on patients is used, maintained or transferred through this system.

Currently, the Diagnosticat’s database is composed of data from 4 influenza seasons. Information is available starting on 2008 and is updated weekly since then. Data is presented as incidence rates per 10^5 ^population, a unit that allows comparison of diagnoses over different territories independently of the number of inhabitants. In this work, the EW is a group of seven days that begins on a Sunday and ends on a Saturday.

Although communicable diseases are in general yearly represented, influenza is represented by seasons due to the characteristics of the influenza epidemic. The epidemic usually starts at the end of the year and ends mid of the following year, peaking in December and January. For this reason and to be consistent with the influenza epidemic, Diagnosticat uses graphics by seasons beginning the EW 23 and ending at EW 22 the following year.

### Statistical data analysis

In this section we aim at obtaining a statistical model for the recorded data of influenza cases. Particularly, we find out that an exponential distribution might be an appropriate way of modeling non-epidemic data. Let us consider that the rate of influenza cases in non-epidemic periods is a random variable *X* and that a database contains weekly realizations of such variable. We define *χ*_*t *_as the set containing chronologically-ordered observations up to time *t*, $\chi_{t}=\{x_{1},\ldots ,x_{t}\}\in R^{+}$. Let us consider that, under the hypothesis that influenza is not active, *X* is exponentially distributed [14], with probability density function (PDF) given by

(1) $$f_{X}(x;\lambda )=\lambda e^{-\lambda x},\;x\geq 0\;$$

and 0 otherwise. Mean and variance are expressed in terms of the parameter *λ *> 0 as E{X}=1/λ and Var{*X*} = 1/*λ*^2^, respectively [15]. Recall that only a subset of *χ*_*t *_follows (1), that is those observations taken in non-epidemic weeks. We define this subset as *ζ*_*t *_⊂* χ*_*t*_ and its elements $\left\{{\tilde{x}}_{1},\ldots ,{\tilde{x}}_{L_{t}}\right\}$ are those from *χ*_*t *_which were detected (using the herein proposed method for instance) as in non-epidemic periods. We use *L*_*t*_ to denote the total number of non-epidemic weeks up to *t*.

Such statistical characterization means that, if not in an epidemic scenario, the rate of cases would be most likely close to zero and decreasing according to the exponential factor *λ*. The factor *λ* is unknown and thus has to be estimated for a complete characterization of (1). In the sequel, we derive the Maximum Likelihood (ML) estimator of *λ* given the random set *ζ*_*t*_. Assuming that observations are independent, the likelihood function of *λ* given *ζ*_*t*_ is

(2) $$ℒ(\lambda )=\lambda^{L_{t}}e^{-\lambda \overset{L_{t}}{\underset{\ell =1}{\sum }}{\tilde{x}}_{\ell }}\;,$$

whose optimization is equivalent to maximization of the log-likelihood. Derivative of the latter yields to

(3) $$\frac{d\ln ℒ(\lambda )}{d\lambda }=\frac{L_{t}}{\lambda }-\overset{L_{t}}{\underset{\ell =1}{\sum }}{\tilde{x}}_{\ell }$$

and equating to zero we obtain the ML estimate of the exponential factor as

(4) $${\hat{\lambda }}_{t}={\left(\frac{1}{L_{t}}\overset{L_{t}}{\underset{\ell =1}{\sum }}{\tilde{x}}_{\ell }\right)}^{-1}\;,$$

which is unbiased and asymptotically consistent with variance $\mathrm{Var}\{{\hat{\lambda }}_{t}\}≃\mathrm{Var}{\left\{X\right\}}^{3}/L_{t}$. Notice that ${\hat{\lambda }}_{t}$ is computed using the *L*_*t *_observations in the non-epidemic period up to the current week *t*.

**Figure 1:**
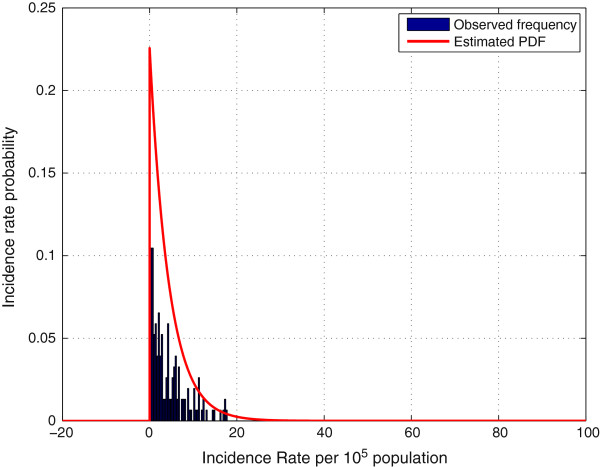
Comparison of sample histogram and the estimated exponential PDF used to model the rate.

We study the goodness-of-fit of the statistical model in our case study, i.e. the database in Diagnosticat. Figure 1 shows a comparison of the histogram of non-epidemic influenza incidence rates and the assumed exponential distribution, with $\hat{\lambda }=4.6899$ estimated as in (4). In order to provide more observations to evaluate the goodness of the exponential fitting, in this case we used all records in the database and omitted those observations that where considered *a posteriori* in the influenza epidemic phase. To identify these data we have selected as the epidemic phase the same weeks as those established by the sentinel surveillance system in Catalonia [16], refer to row *Sentinel network* in Table “Start and end of influenza epidemic in EW per season as detected by the method” to identify the periods. However, in an automated surveillance system, *λ* should be estimated sequentially with an initial guess computed using records from the first season, which could be used as a training sequence. We also run a *χ*^2 ^goodness-of-fit test to validate the exponential assumption, obtaining a *p*-value of 0.8451.

### Sequential influenza detector

The objective is to build an autonomous detection algorithm that is able to determine whether influenza is active or not based on the records of influenza cases. In the case of Diagnosticat, these observations are weekly received, and thus a week-by-week detection is provided by the method. As mentioned in [17], such an automated surveillance system needs to fulfill two objectives: timeliness, to ensure that intervention protocols to the epidemic are in time; and sensitivity, to avoid time/effort wasting due to precipitated measures.

The detector is based on the idea that we have a statistical characterization of the influenza incidence rates when not in the epidemic phase. As discussed earlier, these observations follow an exponential distribution with parameter *λ *estimated as in (4) in batch processing or as in (8) for the sequential treatment of data.

For a given instant *t*, the null hypothesis $H_{0}$ is that the observation *x*_*t*_ comes from a distribution as specified in (1) with ${\hat{\lambda }}_{t-1}$, being the ML estimation at time *t*−1 of the exponential factor. If the hypothesis $H_{0}$ is not accepted, it means that influenza is detected at the *t*-th EW. Equivalently, we say that

(5) $$H_{0}:x_{t}∼f_{X}(x;\lambda ={\hat{\lambda }}_{t-1})\;.$$

We notice that the test has to be conducted using a single observation, *x*_*t*_, from the random variable and that the baseline distribution is continuous and completely specified. Although other alternatives might apply, in this type of decision problems we could obtain enhanced performance by resorting on Empirical Density Function (EDF) statistics to assess $H_{0}$, as claimed in [18]. Since we have only one observation available, the EDF is directly ${\hat{F}}_{X}(x)=1_{\left\{x_{t}\leq x\right\}}$, where 1_{*A*}_ is the indicator function of an event *A*, equal to 1 if *A* is true and 0 otherwise. Here we use the classical Kolmogorov-Smirnov (KS) nonparametric test [19], which is based on the maximum absolute difference between the EDF and the Cumulative Density Function (CDF) of the reference distribution. The latter is known to be *F*_*X*_(*x*;*λ*) = 1−*e*^−*λx*^ for the exponential distribution.

In general, the one-sample KS statistic is defined as

(6) $$D\left({\hat{F}}_{X}(x),F_{X}(x)\right)=\underset{x}{\text{sup}}\left|{\hat{F}}_{X}(x)-F_{X}(x)\right|\;,$$

although in our case the statistic *D* can be simplified accounting that only one observation *x*_*t*_ is used to construct the EDF, and thus the EDF can only take values 0 or 1:

(7) $$D\left({\hat{F}}_{X}(x),F_{X}(x)\right)=\text{max}\left\{1-e^{-\lambda x},e^{-\lambda x}\right\}\;,$$

where we used that |0−*F*_*X*_(*x*)| = |0−1 + *e*^−*λx*^| = 1−*e*^−*λx *^when ${\hat{F}}_{X}(x)=0$.

With this detector, hypothesis $H_{0}$ is rejected at a significance level 0 ≤* α *≤ 1 if *D *≥* γ*_*α*_, where the threshold satisfies $\alpha =P\left\{D>\gamma_{\alpha }\right\}$. Relevant threshold values can be consulted from tables [20,21] if *F*_*X*_(*x*) is completely specified, and from [22] if its parameters should be estimated from data. Notice that the latter is the case here, since *λ* is estimated.

The result of the hypothesis test is binary. Another useful way of reporting the result of the test are *p*-values. Given *d*, a realization of the random variable *D*, we recall that the *p*-value is defined as the probability of obtaining another realization of the test at least as extreme as *d* conditional on $H_{0}$ being true, mathematically $p=P\left\{D\geq d|\;H_{0}\right\}$ (or Type I error). Therefore, not only $H_{0}$ is rejected if the *p*-value of the test is less than *α*, but *p* is also quantifying the *confidence* in such decision. Large *p*-values indicate that *x*_*t*_ is likely to be generated from *F*_*X*_(*x*), and viceversa.

To complete the sequential detection algorithm, we need to estimate the exponential factor *λ* of the baseline distribution. Here we are interested in sequential treatment of epidemic data, and therefore we would like to use an alternative expression to the batch approach in (4) that allows us to compute it recursively instead of processing the entire set of *L*_*t *_non-epidemic weeks each time a new observation is recorded. The propose procedure is applied after the current observation *x*_*t*_ is processed by the KS-based detector. If based on *x*_*t*_ the method rejects $H_{0}$, then we keep the same estimation as previously, ${\hat{\lambda }}_{t}={\hat{\lambda }}_{t-1}$. Otherwise, if *x*_*t *_shows evidences that at the *t*-th EW influenza was active, then we count another week in *L*_*t*_ and use the following recursive expression

(8) $${\hat{\lambda }}_{t}^{-1}=\frac{1}{L_{t}}\left({\hat{\lambda }}_{t-1}^{-1}(L_{t}-1)+x_{t}\right)\;,$$

which is algebraically equivalent to (4). Using (8) instead of (4) has the advantage that new data is processed upon arrival, and thus there is no need to store the complete dataset nor reprocess all data for each new measurement. Initialization of the exponential factor ${\hat{\lambda }}_{0}$ could be computed for instance based on expert, a priori knowledge. In our implementation of the method, we used the non-epidemic weeks in the first influenza season as a training sequence to estimate the value of ${\hat{\lambda }}_{0}$. *L*_*t*_ counts the number of times the null hypothesis was assessed valid up to *t*, and thus it is also the amount of observations used to estimate *λ*.

**Figure 2:**
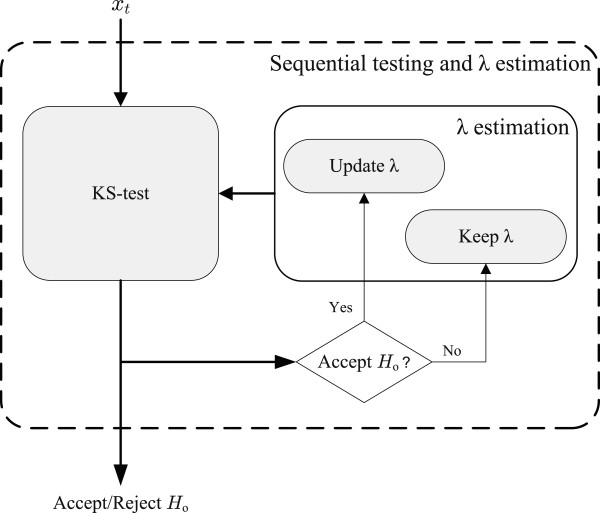
Schematic representation of the sequential method.

The pseudo-code of the sequential influenza detector can be consulted in the Algorithm. Recursive exponential fitting has also been included, note that ${\hat{\lambda }}_{t}$ is updated only when $H_{0}$ is accepted. For the sake of clarity, Figure 2 depicts a block diagram representation of the method. To sum up, when a new observation *x*_*t *_is recorded, it is tested against the exponential distribution and a new value of the statistic *D* is computed which is used to accept or reject $H_{0}$. The parameter of the exponential distribution at the null hypothesis is computed based on all previous observations which were not in epidemic phase, and thus we write ${\hat{\lambda }}_{t-1}$. This estimate is only update according to (8) if $H_{0}$ is accepted based on the current observation, otherwise the previous estimate is kept.

### Algorithm Sequential detection of influenza epidemics

1: Initialization *t *= 1, ${\hat{\lambda }}_{0}$, and *L*_0 _= 1

2: At time *t* new data becomes available, *x*_*t*_

3: Compute statistic $D=\text{max}\left\{1-e^{-{\hat{\lambda }}_{t-1}x_{t}},e^{-{\hat{\lambda }}_{t-1}x_{t}}\right\}$

4: **if***D *≥* γ*_*α *_**then**

5: Reject the null hypothesis ⇒ Flu detected at the *t*-th EW.

6: Keep ${\hat{\lambda }}_{t}={\hat{\lambda }}_{t-1}$ and *L*_*t *_=* L*_*t*−1_

7: **else if***D *<* γ*_*α*_** then**

8: Accept the null hypothesis ⇒ Flu not detected at the *t*-th EW.

9: Set *L*_*t *_=* L*_*t*−1_ + 1, the number of observations used to calculate ${\hat{\lambda }}_{t}$

10: Update ${\hat{\lambda }}_{t}^{-1}=\frac{1}{L_{t}}\left({\hat{\lambda }}_{t-1}^{-1}(L_{t}-1)+x_{t}\right)$

11: **end if**

12: *t *=* t* + 1 and go to step 2

## Results and discussion

We used the open database described earlier to test the detector. We also validated in this experiment the proposed ML data fitting that characterizes non-epidemic cases as exponentially distributed. At this time of writing, we had available data from the 2008-2009 to the 2011-2012 seasons.

The parameter *λ* was estimated sequentially as in (8). Initially, this value was calculated using data from the first influenza season (i.e., 2008-2009), as a training sequence for ${\hat{\lambda }}_{0}$, with those observations identified by the Catalan sentinel network as being in the epidemic phase [16] removed from that year’s set *χ* to form the subset *ζ*. In particular, incidence rates per 10^5^population above 20 were not considered. If not otherwise stated, we used 20/10^5^ to train the method, although later in this section we provide a sensitivity analysis with respect to this threshold. The resulting exponential factor was ${\hat{\lambda }}_{0}=3.8617$ for the first year, which was updated as described in the algorithm when new data from EW 23 of the influenza season 2009-2010 was available.

**Figure 3:**
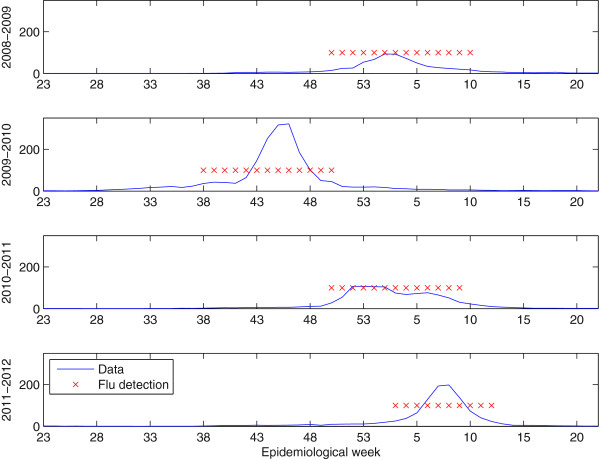
**Weekly influenza-detection results for 4 influenza seasons along with recorded data. *y*-axis represents the influenza cases per 10**^**5**^**inhabitants (solid blue) and the detected influenza outbreaks output (red crosses).**

We tested the observation corresponding to the *t*-th EW according to (7) with a significance level *α *= 0.05. The results of the test (red crosses) and the registered influenza incidence rates (blue solid line) used to infer the decision are plotted in Figure 3. Red crosses denote the EW where $H_{0}$ was rejected, i.e. influenza activity was detected. The test detected the following weeks as epidemic for each influenza season: 50−10 (2008-2009 season), 38−50 (2009-2010 season), weeks 50−9 (2010-2011 season) and weeks 3 to 12 for the current 2011-2012 season. The results obtained were in accordance to the assessment provided by the official system of influenza surveillance in Catalonia (PIDIRAC) [16,23-25]. PIDIRAC establishes the epidemic periods after the analysis of data from sentinel network and virus activity (laboratory surveillance data). In Table 1 we summarize the results in terms of start and end of influenza periods per season. Comparing the obtained results to those given by the official source we can calculate for the method an accuracy of 90%, a sensitivity of 100% and a specificity of 87.6% for the complete four seasons. Recall that we used a threshold on the first season of 20/10^5^ to estimate ${\hat{\lambda }}_{0}$ based on [16]. The selection of this value needs to be done depending on the application. For the sake of completeness, the table also shows a sensitivity analysis to such threshold. The conclusion is that if this parameter is set within reasonable intervals (as discussed earlier, based on prior knowledge), the results of the detector are not drastically varying. Indeed, the most sensitive season is the first, which is used for ${\hat{\lambda }}_{0}$ calculation. Detection results for other seasons remain unaltered. Increasing the threshold makes the method less sensitive to epidemic data, and viceversa.

**Table 1:** Start and end of influenza epidemic in EW per season as detected by the method

	**2008-2009**		**2009-2010**		**2010-2011**		**2011-2012**	
**Threshold**	Start	End	Start	End	Start	End	Start	End
10/10^5^	50	11	38	50	50	9	3	12
20/10^5^	50	10	38	50	50	9	3	12
30/10^5^	51	8	38	50	50	9	3	12
40/10^5^	52	7	38	50	50	9	3	12
50/10^5^	52	7	38	50	50	9	3	12
**Sentinel network**	51	8	41	51	51	9	4	12

Sensitivity to a number of thresholds on the first season to estimate ${\hat{\lambda }}_{0}$ is reported. Also, the official reports based on the sentinel network are included.

It is important to notice that in the influenza season 2009-2010, the A(H1N1) Influenza virus pandemic occurred during autumn in Catalonia, fact that caused a different temporal pattern of influenza epidemics [26]. Recall that similar differences were reported worldwide [27,28]. That epidemic had higher incidence rates than seasonal influenza and took place some weeks before. The proposed method was also able to cope with this non-seasonal event as its departure from $H_{0}$ was similar to that of seasonal influenza.

**Figure 4:**
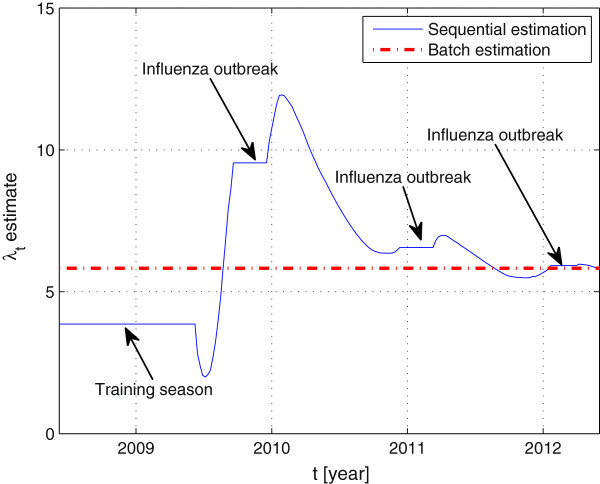
**Estimation of the exponential factor *λ *using batch and sequential approaches.**

Figure 4 shows the evolution over time of ${\hat{\lambda }}_{t}$, where the initial training can be observed as the constant value $\hat{\lambda }=3.8617$. It becomes clear from this figure that the sequential updating is only performed in non-epidemic periods, and constant otherwise. For the sake of completeness, we compared the results of the sequential method to the estimates obtained after processing all data using the batch method in (4). Notice that both methods provide the same estimate after all data is processed, with the advantage that the sequential procedure does not require storage of all observations before processing them.

**Figure 5:**
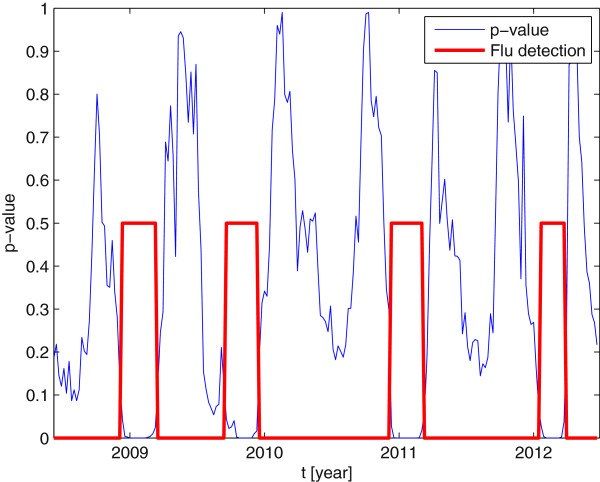
**Sequence of *p*-values obtained by the detector.**

In order to provide more insight in the behavior of the detector, Figure 5 shows the obtained *p*-values. As commented earlier, this value quantifies how likely the null hypothesis is true. We can observe that low values were obtained when $H_{0}$ was rejected and that it grew in non-epidemic periods. In the latter, *p*-values were not very large (ideally close to one) due to very small sample size, recall that we were constrained to use a single observation per EW to infer the decision. It is remarkable that, despite the small sample size, results were in accordance to what the official system based on the sentinel network recorded.

Indeed, the proposed method can be used in general to the detection of other infectious diseases whose statistical characterization is available. If non-epidemic periods could be characterized by an exponential distribution, the usage of the method is straightforward. Otherwise, if another distribution better fits the data, slight modification of the method should be performed to compare it with the EDF and to update the required parameters of the PDF. A limitation of the method is related to the data gathering method, which has to be continuously recording observations. Since the method is based on non-epidemic data to estimate the distributional parameters and to assess whether the disease is active or not, some systems like sentinels cannot straightforwardly benefit from this tool. Recall, for instance, that sentinels are likely to stop recording data in typically inactive periods of the disease, these inactive periods have an impact in the quality of the estimated distribution of non-epidemic data.

## Conclusions

In this paper we proposed an automated method to detect influenza outbreaks from periodically recorded incidence rates. In contrast to setting yearly predefined thresholds to determine influenza outbreaks by data inspection, we presented a detector based on the statistical properties of non-epidemic data. The method can be useful to complement traditional surveillance methods. The algorithm provides a binary signal indicating epidemic activity as well as a quantitative measure (i.e., the *p*-value) of its confidence. The method can be executed sequentially and it is self-adjusted (after some initialization of the exponential factor). We presented results with real medical data from Catalonia, showing the performance of the detector even in non-seasonal scenarios, as well as the validity of the exponential modeling for non-epidemic influenza incidence rates. Finally, the objectives required for such an automated system are seen to be fulfilled: timeliness and sensitivity.

Timeliness is generally defined as the difference between the time an event occurs and the time the reference standard for that event occurs. Diagnosticat data is available the instant the epidemiologic week has endend, and thus advances in four days the publication of data over the sentinel network based system in Catalonia. Additionally, the proposed sequential detection method signals the event 1 week earlier on average for the tested data (excluding the AH1N1.pdm.2009 season).

## Endnote

^a^ Note that in this work we use *on-line* to denote that the algorithm operates *sequentially*, that is, as new data becomes available.

## Abbreviations

CDF: Cumulative Density Function; CLT: Central Limit Theorem; CTTC: Centre Tecnològic de Telecomunicacions de Catalunya; eCAP: Electronic health record in Catalonia; EDF: Empirical Density Function; EW: Epidemiological week; ICS: Catalan Institute of Health; KS: Kolmogorov-Smirnov; ML: Maximum Likelihood; PDF: Probability Density Function; SISAP: Information Systems for Primary Care Services; WHO: World Health Organization.

## Competing interests

The authors declare that they have no competing interests.

## Authors’ contributions

PC designed the algorithm, performed the analysis and drafted the manuscript. EC and LM provided the data and helped draft the manuscript. All authors have read and approved the final manuscript.

## Pre-publication history

The pre-publication history for this paper can be accessed here:

http://www.biomedcentral.com/1472-6947/12/112/prepub
